# Promising Treatment Strategy for Primary Malignant Melanoma of the Esophagus by Radical Esophagectomy and Nivolumab as Adjuvant Therapy: A Case Report

**DOI:** 10.70352/scrj.cr.25-0027

**Published:** 2025-05-09

**Authors:** Yusuke Yamamoto, Junya Kitadani, Keiji Hayata, Taro Goda, Shinta Tominaga, Tomoki Nakai, Shotaro Nagano, Ryuta Iwamoto, Manabu Kawai

**Affiliations:** 1 Second Department of Surgery, Wakayama Medical University, Wakayama, Wakayama, Japan; 2 Department of Human Pathology, Wakayama Medical University, Wakayama, Wakayama, Japan

**Keywords:** esophagectomy, malignant melanoma, nivolumab, adjuvant therapy

## Abstract

**INTRODUCTION:**

Primary malignant melanoma of the esophagus (PMME) is a rare malignant tumor of the esophagus with very poor prognosis due to high rates of recurrence and metastasis even after radical resection. Recently, however, immune checkpoint inhibitors such as anti-programmed cell death-1 antibodies have been suggested to improve the prognosis of malignant melanoma. This report describes the use of postoperative nivolumab as adjuvant therapy after surgical resection of PMME, with recurrence-free follow-up for more than 1 year.

**CASE PRESENTATION:**

A 69-year-old man had chest discomfort and tightness in his throat. Upper gastrointestinal endoscopy revealed multiple melanosis and an elevated lesion in the middle esophagus. After histological examination, he was diagnosed as having PMME, so he underwent thoracoscopic subtotal esophagectomy, three-field lymphadenectomy, pedunculated jejunum reconstruction with super-charge and super-drainage, and feeding jejunostomy due to the past history of gastrectomy. The adjuvant therapy using nivolumab (every 2 weeks, 240 mg) for 1 year was completed with no serious side effects, and there was no recurrence for more than 1 year postoperatively.

**CONCLUSIONS:**

Although cases of PMME treated with adjuvant nivolumab have rarely been reported, the present case suggests that this approach may represent a promising treatment option, similar to cutaneous melanoma.

## Abbreviations

PD-1
: programmed cell death-1
PET
: positron emission tomography
PMME
: primary malignant melanoma of the esophagus
SOX
: SRY-related HMG-box

## INTRODUCTION

PMME is a malignant tumor originating from melanocytes in the basal portion of the esophageal squamous epithelium and the stromal border. PMME is rare, accounting for <0.1%–0.2% of all esophageal malignancies, and <0.05% of all melanoma subtypes.^1)^ Poor prognosis of PMME is associated with delayed detection and a high likelihood of metastasis and recurrence. Esophagectomy is a radical treatment for PMME, but the recurrence rate remains high. Furthermore, the prognosis has been extremely poor, with an average life expectancy of 13.5 months at the time of diagnosis.^2)^ However, with the improvement of diagnostic techniques for early detection and the use of immune checkpoint inhibitors such as anti-PD-1 antibodies, the prognosis is considered to be improved.^3–6)^ Cases of long-term survivors who underwent immune checkpoint inhibitors for PMME have been reported.^7–15)^ However, the number of such cases remains limited, and further research is needed to provide conclusive evidence. In this report, we describe a case of PMME treated with radical esophagectomy followed by adjuvant therapy with nivolumab that has been relapse-free for more than 1 year. Furthermore, literature reviews regarding nivolumab for the treatment of PMME were performed.

## CASE PRESENTATION

A 69-year-old man with a history of pyloric gastrectomy for duodenal ulcer, multiple colonic polyps and hemorrhoids saw his usual doctor with chest discomfort and tightness in his throat. Upper gastrointestinal endoscopy revealed melanosis and an elevated lesion in the middle esophagus, so he was referred to our department. There was no pigmentation of the skin or oral mucosa.

**Fig. 1:**
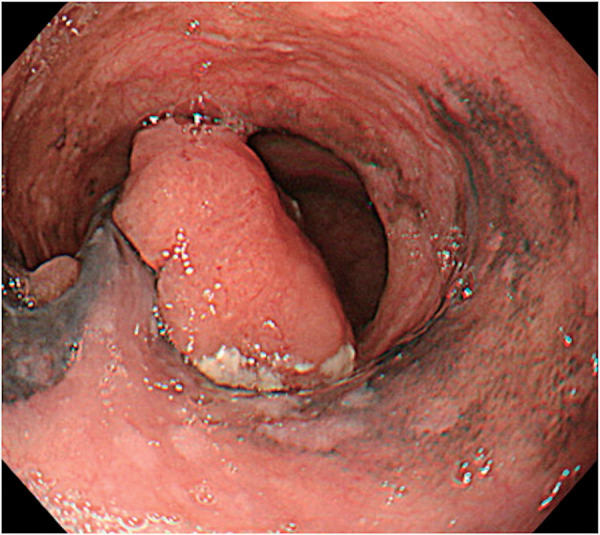
Esophagogastroscopy showed a 40 mm large dark brown tumor with a surrounding 100 mm black pigmentation, located in the middle thoracic esophagus.

**Fig. 2:**
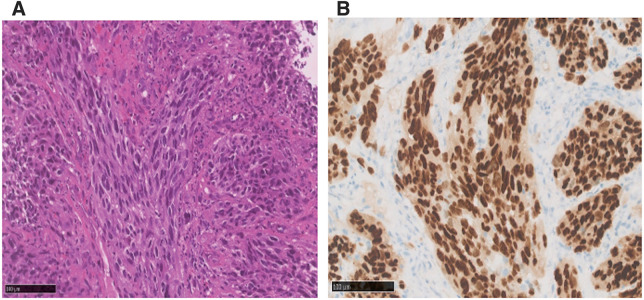
Microscopic findings of the biopsy specimen. (**A**) Histopathological examination with magnification ×20. (**B**) SOX-10 staining with magnification ×20.

**Fig. 3:**
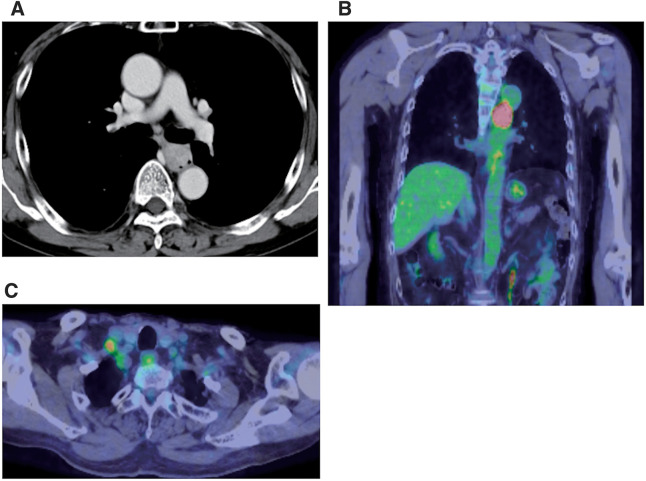
Preoperative findings. (**A**) Contrast-enhanced computed tomography of chest showing a mass lesion with contrast effect in the middle thoracic esophagus. (**B**) Fluorodeoxyglucose positron emission tomography-computed tomography showing an abnormal accumulation in the same region. (**C**) Accumulated lymph node (Station No. 104R).

Re-examination of upper gastrointestinal endoscopy showed a 40 mm large, dark brown tumor with a surrounding 100 mm black pigmentation in the middle thoracic esophagus (**Fig. 1**). Tumor biopsy revealed proliferation of polygonal and spindle-shaped cells with reduced adherence, prominent nucleoli and stromal fibrosis (**Fig. 2A**). Immunostaining was negative for CK (AE1/AE3), p63, and S100, but diffusely strongly positive for SOX 10, which confirmed diagnosis of PMME (**Fig. 2B**). PD-L1 protein (clone 28-8) was expressed in <1%. Contrast-enhanced CT scan showed a mass lesion with contrast effect in the middle thoracic esophagus (**Fig. 3A**) and an 8 mm-sized lymph node swelling (Station No. 104R). There was no obvious distant metastasis. Fluorodeoxyglucose PET-CT indicated abnormal accumulation in each region (**Fig. 3B** and **3C**).

**Fig. 4:**
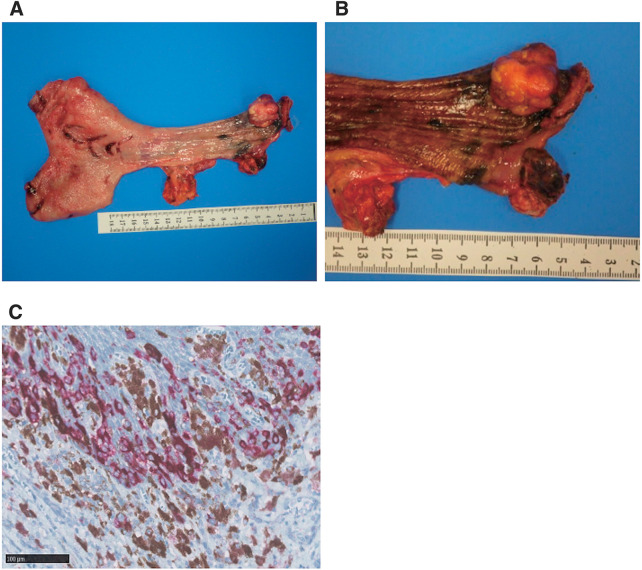
Surgical specimen of esophagus. (**A**) Protruding into the lumen was formed at 10 mm from the proximal end. (**B**) Blackish changes in the surrounding mucosa. (**C**) Microscopic findings of the surgical specimen of Melan-A staining with magnification ×20.

The patient was diagnosed as cStage IVa PMME (cT2, cN0, cM1a [AJCC 8th^16)^]) and underwent thoracoscopic subtotal esophagectomy, 3-field lymphadenectomy, pedunculated jejunum reconstruction with super-charge and super-drainage, and feeding jejunostomy. The operation time was 661 minutes and the blood loss was 420 mL. Minor leakage occurred at the esophagojejunostomy (Clavien-Dindo classification Grade II). The patient was discharged 50 days after the surgery. A nodular mass (25 × 22 mm) protruding into the lumen had formed 10 mm from the proximal cut end and blackish changes were observed in the surrounding mucosa (**Fig. 4A** and **4B**). Pathologically, atypical spindle-shaped cells proliferated mainly in bundles, and atypical epithelial-like cells producing brown pigment proliferated solidly on the basal epithelial layer of the surrounding mucosa, indicating PMME (pT3, LVI [−], PMx, pDM0, pRM0, pN0). Preoperative PET-CT showed uptake of the Standard Uptake Value max 4. Lymph node dissection on both sides of No. 104 was completely performed, but no metastases were observed in the pathological findings. Therefore, swollen lymph nodes were considered to be inflammatory changes. SOX-10 and Melan A positive melanocytes (**Fig. 4C**) were distributed up to 1.5 mm from the cut edge, and melanin deposition and melanocyte proliferation were observed at the cut edge beyond the melanocyte distribution. Although melanocytes were found at the resection margin, the degree of cellular atypia was mild. Finally, they were diagnosed as ectopic melanocytes and designated as PMx. With the patient’s informed consent, nivolumab therapy (240 mg, every 2 weeks) was administered for 1 year. The patient showed no serious adverse events related to immunotherapy and is alive without relapse at the point of completion of adjuvant therapy. Follow-up findings of contrast-enhanced CT and esophagogastroscopy after completion of adjuvant therapy showed no recurrence of melanoma (**Supplementary Fig. 1**).

## DISCUSSION

PMME is a rare tumor of the esophagus, with only about 480 cases reported by 2021 since Baur reported the first case in 1906.^17)^ Due to its rare occurrence, the clinical and molecular etiology and the optimal treatment of PMME are not properly understood. The prognosis of these tumors has been considered poor, with a reported 5-year survival rate of 4% in resected cases.^18,19)^ However, recent advances in endoscopic techniques have enabled early diagnosis, and a recent paper reported the 5-year survival rate after resection as 37%.^20)^

The average age of onset of PMME is 60 years, younger than that of esophageal cancer, and dysphagia and epigastralgia are common chief complaints.^21)^ The middle and lower esophagus account for 92.1% of all cases and more than half have invasion deeper than the muscular layer when detected, which is also thought to be a factor in the poor prognosis.^22)^ Surgical resection is the most common treatment and according to one report, 59 of 76 patients with PMME (77.6%) underwent esophagogastrostomy with subtotal esophagectomy and lymph node dissection, with subsequent recurrence identified in 89.7% of patients postoperatively.^22)^ In another report, more than 90% of postoperative recurrences in patients with PMME occurred within 2 years after the primary surgery.^2)^ The most frequent sites of recurrence were reported to be lymph nodes, the liver, the lungs, in bone, and in sites of anastomosis.^2)^

The high recurrence rate dictates the need for postoperative adjuvant therapy, but there is no consensus on the use of postoperative chemotherapy for rare cancers.^22–24)^ The combination of temozolomide and cisplatin as adjuvant chemotherapy for malignant melanoma significantly improved both relapse-free survival and overall survival.^22)^ However, the CheckMate 066 study for patients with unresectable, untreated stage III and stage IV malignant melanoma compared nivolumab with the conventional chemotherapy of dacarbazine.^25)^ Nivolumab had significantly improved response rate, progression-free survival, and overall survival. Nivolumab has thus become a standard treatment for advanced malignant melanoma. The CheckMate 238 trial also showed that nivolumab significantly prolonged relapse-free survival compared with the cytotoxic T-lymphocyte antigen-4 inhibitor ipilimumab in Stage IIIB or IIIC and IV malignant melanoma after resection.^26)^ Additionally, the CheckMate 76 K revealed a statistically significant improvement in 1-year recurrence-free survival with nivolumab versus placebo in Stage IIB/C melanoma (89% vs. 79%; HR 0.42, 95% CI: 0.30–0.59; P < 0.0001), underscoring its role in reducing recurrence risk by nivolumab for 12 months.^27)^ As with this report, a 1-year course of adjuvant nivolumab is considered appropriate for PMME.

Malignant melanoma is characterized by a high tumor mutational burden and a diversity of tumor-associated antigens, contributing to recognition by the immune system and making it highly responsive to immune checkpoint inhibitors.^28)^ As with cutaneous melanoma, PMME is expected to be a target for immune checkpoint inhibitors. Furthermore, the CheckMate 577 trial demonstrated the efficacy of nivolumab as adjuvant therapy for esophageal or gastroesophageal junction cancer, showing a significant improvement in disease-free survival compared with a placebo.^29)^ This landmark result supports the potential use of nivolumab in other malignancies, including rare cancers such as PMME. Due to the aggressive nature of PMME and the limited options available for adjuvant therapy, the concept of using nivolumab as adjuvant treatment in this setting appears to be promising, although there are currently no large-scale trials specifically designed for PMME. However, extrapolation of the CheckMate 577 results to PMME could guide future investigations and therapeutic strategies.

Only 13 patients with PMME who underwent administration of nivolumab have been reported, including our case (**Table 1**).^7–15,30–32)^ In these reports, nivolumab was primarily used for PMME with recurrence or metastasis, although one case involved its use as neoadjuvant therapy. Additionally, only three cases, including ours, reported the use of nivolumab as adjuvant chemotherapy for PMME, with no recurrences reported to date.^9,15)^ Similar to cutaneous melanoma, our results suggest that radical esophagectomy followed by 1 year of adjuvant therapy with nivolumab may be a promising treatment strategy for PMME in the future. While nivolumab has been suggested to improve progression-free survival in cases of metastatic or recurrent PMME, its efficacy as an adjuvant therapy for PMME has not yet been proven. Validation studies of adjuvant therapy with nivolumab for PMME are needed to collect more cases and long-term follow-up data, although the number of patients is limited due to rare cancer types.

**Table 1:** Characteristics of patients treated with nivolumab as adjuvant or systemic chemotherapy for PMME

No.	Author	Year	Age	Sex	Stage (AJCC/UICC 8th edition)	Metastasis or recurrence	Therapy	Therapeutic duration	Adverse events	Follow-up period	Outcome
1	Inadomi et al.^31)^	2016	73	M	IV	Liver, lung, lymph node	Chemoradiotherapy → nivolumab	1 month (Chemoradiotherapy) → 4 months	Bi-cytopenia	7 months	Dead
2	Endo et al.^7)^	2020	70	M	Ⅲ	Retroperitoneal	Nivolumab	41 courses	Adrenal insufficiency	20 months	Alive
3	Ito et al.^11)^	2021	81	F	Ⅲ	Multiple lymph nodes, bone	Nivolumab	30 courses	None	27 months	Dead
4	Yamaguchi et al.^14)^	2021	75	F	Ⅱ or Ⅲ	Pleural dissemination, pancreas	Nivolumab → RT	3 courses → RT	Renal dysfunction	26 months	Alive
5	Tsukamoto et al.^13)^	2021	74	M	Ⅰ	Liver	Nivolumab → nivolumab + ipilimumab	7 courses → nivolumab + ipilimumab 2 courses	None	Unknown	Unknown
6	Okamoto et al.^10)^	2021	79	M	IV	Lymph nodes, lung, pleural, peritoneal metastases	Nivolumab	6 courses	Kidney injury	5 years	Alive
7	Kashfi et al.^32)^	2022	76	F	IV	Bone	Nivolumab	2 courses	None	2 months	Dead
8	Hanada et al.^8)^	2022	65	F	Ⅰ	Jejunum, cervical esophagus, lymph nodes, lung	Nivolumab	32 courses	None	9 months	Alive
9	Furune et al.^30)^	2022	62	M	II or III	None	Nivolumab	7 months	Interstitial pneumonitis	Unknown	Unknown
10	Nambara et al.^9)^	2022	60	F	Ⅳ	None	Nivolumab	1 year (adjuvant)	Pneumothorax	12 months	Alive
11	Attlassy et al.^12)^	2023	66	F	IV	Liver	Pembrolizumb → nivolumab + ipilimumab	Pembrolizumb 8 courses → nivolumab + ipilimumab 4 months	Kidney disease	3 years	Alive
12	Shibayama et al.^15)^	2025	75	M	III	None	Ipilimumab and nivolumab → operation → nivolumab	Ipilimumab and nivolumab 4 courses (neoadjuvant) → one year (adjuvant)	Hyponatremias	3 years	Alive
13	Our case	2025	69	M	Ⅱ	None	Nivolumab	24 courses (adjuvant)	None	12 months	Alive

RT, radiation therapy

## CONCLUSIONS

Postoperative adjuvant therapy with nivolumab for PMME seems to be a safe, feasible, and potentially effective treatment to improve long-term outcomes. However, further case series and validation are needed.

## SUPPLEMENTARY MATERIALS

Supplementary Fig. 1 Follow-up findings. (**A**) Contrast-enhanced CT showed no evidence of recurrence. (**B**) Esophagogastroscopy showed no pigmentation at the anastomosis site, suggestive of recurrence of melanoma.

## ACKNOWLEDGMENTS

The authors acknowledge proofreading and editing by Benjamin Phillis from the Clinical Study Support Center at Wakayama Medical University.

## DECLARATIONS

### Funding

Not applicable.

### Authors’ contributions

YY and JK drafted the manuscript.

KH, TG, ST, TN, SN, and MK critically revised the manuscript.

RI gave pathological diagnosis and collected the pathological pictures.

All authors have agreed to and significantly contributed to this case report.

All authors have read and approved the manuscript.

### Availability of data and materials

The data that support the findings of this study are available from the corresponding author upon reasonable request.

### Ethics approval and consent to participate

All procedures performed in studies involving human participants were in accordance with the ethical standards of the institutional and/or national research committee and with the 1964 Helsinki Declaration and its later amendments or comparable ethical standards. We got the consent from the patient to participate in this study.

### Consent for publication

The patient provided permission to publish the features of his case.

### Competing interests

The authors declare that they have no competing interests.
