# Supplementary figures and images for: Promising Treatment Strategy for Primary Malignant Melanoma of the Esophagus by Radical Esophagectomy and Nivolumab as Adjuvant Therapy: A Case Report
Source: Surg Case Rep. 2025 May 9;11(1):25-0027. doi: 10.70352/scrj.cr.25-0027 (PMC12068939; doi:10.70352/scrj.cr.25-0027)

Supplementary Fig. 1

A

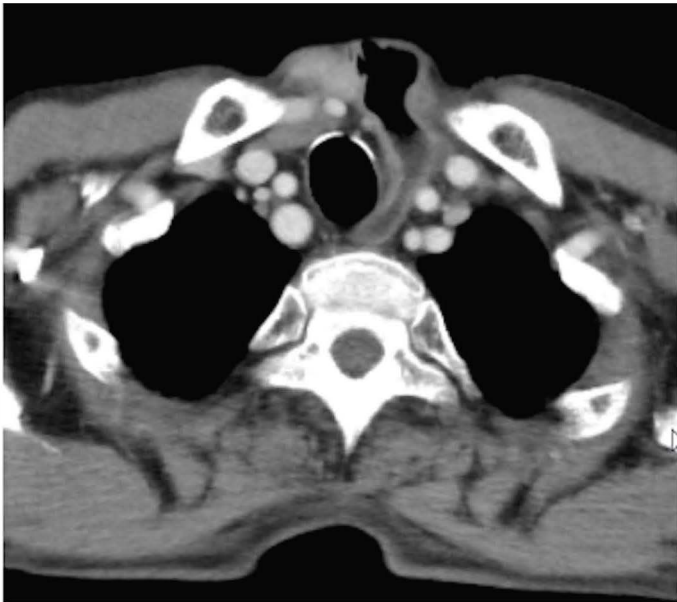

B

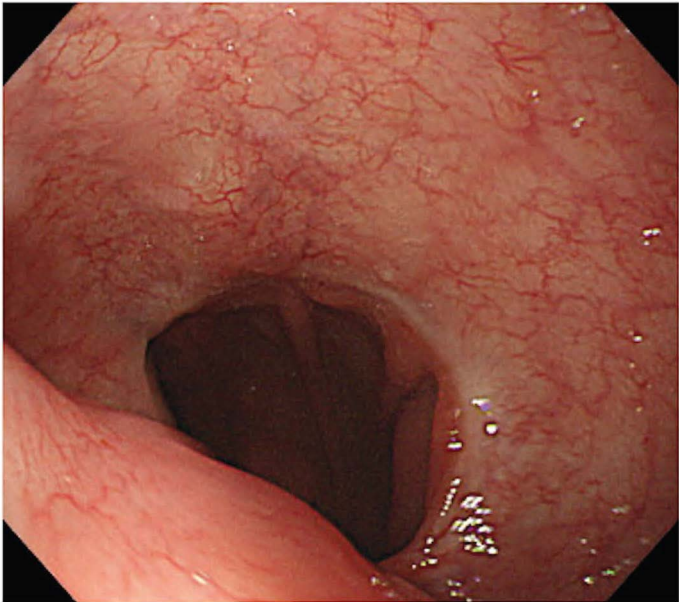

Supplement: Supplementary Fig. 1 [file scr-11-01-25-0027-s001.pdf]
